# Laser Microdissection and Spatiotemporal Pinoresinol-Lariciresinol Reductase Gene Expression Assign the Cell Layer-Specific Accumulation of Secoisolariciresinol Diglucoside in Flaxseed Coats

**DOI:** 10.3389/fpls.2016.01743

**Published:** 2016-11-21

**Authors:** Jingjing Fang, Aïna Ramsay, Sullivan Renouard, Christophe Hano, Frédéric Lamblin, Brigitte Chabbert, François Mesnard, Bernd Schneider

**Affiliations:** ^1^Max Planck Institute for Chemical EcologyJena, Germany; ^2^EA3900 – BioPI Faculté de Pharmacie, Université de Picardie Jules VerneAmiens, France; ^3^Laboratoire de Biologie des Ligneux et des Grandes Cultures, UPRES EA 1207, Antenne Scientifique Universitaire de Chartres, Université d’OrléansChartres, France; ^4^INRA, UMR614 Fractionnement des AgroRessources et EnvironnementReims, France; ^5^UMR614 Fractionnement des AgroRessources et Environnement, Université de Reims Champagne-ArdenneReims, France

**Keywords:** flaxseed, gene expression, laser microdissection, lignans, *Linum usitatissimum*, localization, pinoresinol-lariciresinol reductase, secoisolariciresinol diglucoside

## Abstract

The concentration of secoisolariciresinol diglucoside (SDG) found in flaxseed (*Linum usitatissimum* L.) is higher than that found in any other plant. It exists in flaxseed coats as an SDG-3-hydroxy-3-methylglutaric acid oligomer complex. A laser microdissection method was applied to harvest material from different cell layers of seed coats of mature and developing flaxseed to detect the cell-layer specific localization of SDG in flaxseed; NMR and HPLC were used to identify and quantify SDG in dissected cell layers after alkaline hydrolysis. The obtained results were further confirmed by a standard molecular method. The promoter of one pinoresinol-lariciresinol reductase gene of *L. usitatissimum* (*LuPLR1*), which is a key gene involved in SDG biosynthesis, was fused to a β-glucuronidase (*GUS*) reporter gene, and the spatio-temporal regulation of *LuPLR1* gene expression in flaxseed was determined by histochemical and activity assays of *GUS*. The result showed that SDG was synthesized and accumulated in the parenchymatous cell layer of the outer integument of flaxseed coats.

## Introduction

*Linum usitatissimum*, a plant with multiple uses, has traditionally been cultivated for fiber and oil production. Flaxseed is a rich source of polyunsaturated fatty acids and lignans. These components contribute beneficial nutritional and health-related functions to a flaxseed diet (Oomah, 2001; Adolphe et al., 2010; Singh et al., 2011).

**FIGURE 1:**
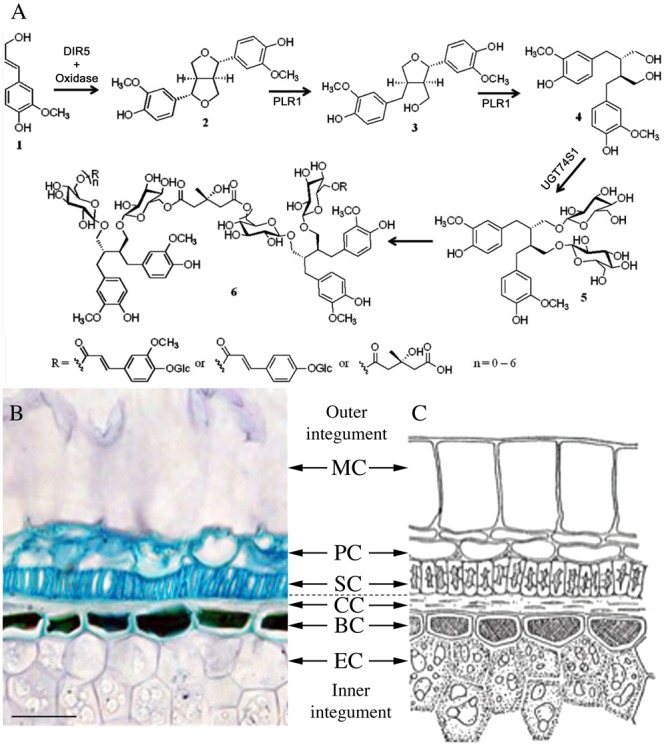
**(A)** Proposed biosynthesis pathway of SDG oligomer in flaxseed (from Ghose et al., 2014 and Dalisay et al., 2015). **(B,C)** Anatomy of a mature flaxseed coat. **(A)** Micrograph of a semi-thin section (5 μm) of mature flaxseed (cv. Barbara) coats stained with toluidine blue. Bar represents 50 μm. **(B)** Schematic organization of mature flaxseed coats (from P. Ozenda, Les végétaux – organization et diversité biologique, Ed. Dunod, 2000 p 378, ISBN 2 10 004684 5), reproduced with kind permission of Dunod Éditeur (Paris, France). MC, mucilaginous cell; PC, parenchymatous cell; SC, sclerified cell; CC, compressed cell; BC, brown cell; EC, endosperm cell.

Flaxseed is by far the richest source of lignans in the plant kingdom. Between 6.1 and 28.8 mg/g of secoisolariciresinol diglucoside (SDG) (**5** in **Figure 1A**), was reported as the predominant lignan in whole flaxseed. This SDG concentration is higher in flaxseed than in any other edible plant (Axelson et al., 1982; Thompson et al., 1991). Free SDG was almost undetectable in flaxseed at any developing stage (Ford et al., 2001; Hano et al., 2006). Directly after its formation, SDG is assembled into oligomeric lignan macromolecules (**6** in **Figure 1A**), in which from one to seven SDGs are linked by 3-hydroxy-3-methylglutaric acid (HMGA) through ester bonds (Klosterman and Smith, 1954; Kamal-Eldin et al., 2001; Struijs et al., 2009). Here, these macromolecules are collectively designated the SDG-HMG oligomer complex.

The following biosynthetic pathway (see **Figure 1A**) of both SDG and SDG macromolecules in flaxseed, has been proposed: a dirigent protein-assisted coupling of two *E*-coniferyl alcohol (**1** in **Figure 1A**) units results in one molecule of (-)-pinoresinol (**2** in **Figure 1A**). (-)-Pinoresinol is successively converted into (-)-lariciresinol and (+)-secoisolariciresinol (**3** and **4** in **Figure 1A**). Then the latter molecule is glycosylated to SDG. Finally, SDG condenses with HMG-CoA to form the SDG-HMG oligomer complex (Umezawa et al., 1991; Ford et al., 2001; Hano et al., 2006; Ghose et al., 2014; Dalisay et al., 2015).

The *L. usitatissimum* pinoresinol-lariciresinol reductase (*LuPLR1*) gene encoding the enzyme responsible for the synthesis of the major enantiomer (+)-secoisolariciresinol is strongly expressed in the seed coats of developing flaxseed, suggesting that SDG is synthesized and accumulated in this tissue (Hano et al., 2006). This is in agreement with enhanced levels of SDG in mechanically obtained coat-enriched fractions of mature seeds (Madhusudhan et al., 2000; Wiesenborn et al., 2003) and with the MALDI mass spectrometry imaging data obtained by Dalisay et al. (2015). RNAi technology applied to down regulate *LuPLR1* gene expression in flax (*Linum usitatissimum* L.) seeds has evidenced the direct *in vivo* implication of the *LuPLR1* gene in the biosynthesis of (+)-SDG (Renouard et al., 2014). *LuPLR2*, another *PLR* gene, encoding a protein with an enantiospecificity opposite to LuPLR1 exists in flaxseeds (Hemmati et al., 2010). *LuPLR2* is responsible for the synthesis of the minor (–)-secoisolariciresinol enantiomer. It is generally accepted knowledge that lignans accumulate in the seed coats and are absent in embryos.

**Figures 1B,C** shows the anatomical structure of mature flaxseed coats. It consists of an outer integument with layers of mucilaginous cells (MCs), parenchymatous cells (PCs), and sclerified cells (SCs) and a flat layer of compressed cells (CCs), and the inner integument comprising the brown cell (BC) and the endosperm cell (EC) layers. A previous study performed in our lab (BIOPI) using antibodies against secoisolariciresinol, the aglycone of SDG, suggested accumulation of SDG mainly in the SCs of the outer integument of mature flaxseed (Attoumbré et al., 2010). The same method was applied to detect SDG in immature flaxseed at different developmental stages (Attoumbré et al., 2011). MALDI mass spectrometry imaging experiments localized SDG and SDG-HMG in the outer integument of developing and mature flax seeds (Dalisay et al., 2015).

Further localization studies to better understand the site of synthesis and accumulation of SDG at a cellular level are presented here. Knowledge of cell-type specific localization of metabolites in seed coats would shed light on the putative ecological function of lignans, especially of the SDG oligomer complex, in defending the seeds against pathogens and/or their physiological role in seed development and germination. In addition, knowing the biosynthetic capacity of special cells in the seed coat would allow studying regulatory processes involved in individual steps of lignan biosynthesis in these cells. Understanding regulation of the lignan biosynthetic pathway would potentially open the chance to establish biotechnological production of lignans.

Laser microdissection (LMD) has been used to harvest specific tissues or cells from plant materials for transcript and protein analysis (Hölscher and Schneider, 2008; Nelson et al., 2008; Rajhi et al., 2011) and enabled micro-spatial metabolic profiling studies (Schneider and Hölscher, 2007; Nakashima et al., 2008; Hölscher et al., 2009). In this work, LMD was employed to sample the materials from different layers of both developing and mature flaxseed coats. After SDG was released by alkaline hydrolysis, NMR and HPLC methods were applied to identify and quantify SDG in these samples by comparing data with those of the standard compound isolated from flaxseed coats.

The *LuPLR1* gene, which is involved in converting (–)-pinoresinol into (–)-lariciresinol and then into (+)-secoisolariciresinol, is expressed in flaxseed coats (Hano et al., 2006). To confirm the results obtained by LMD, flax transgenic plants containing the *LuPLR1* gene promoter upstream from the β-glucuronidase (*GUS*) reporter gene (Jefferson et al., 1987) were prepared. The histochemical staining of *GUS* was used to determine the location of *LuPLR1* gene expression in different developmental stage seeds, as well as *LuPLR1* transcript was detected in different parts of wild type flaxseed by the reverse transcription-polymerase chain reaction (RT-PCR). Moreover, fluorescence spectrophotometry was applied to quantify *GUS* activities in various parts of the flaxseed to obtain temporal information about *LuPLR1* gene expression.

In this article, we describe the cell layer-specific detection of secondary metabolites by using chemical and molecular methods in parallel to elucidate both the spatial distribution of SDG as well as the temporal production of SDG in flaxseed during maturation.

## Materials and Methods

### Wild Type and Transgenic Flaxseed

Seeds of *L. usitatissimum* cv. Barbara were obtained from the cooperative Terre de Lin (Fontaine le Dun, France) and Laboulet semences (Airaines, France). Flax plants used for LMD were raised in the greenhouse of the Max Planck Institute for Chemical Ecology in Jena, Germany. The plants were grown in soil under greenhouse conditions (day 22–24°C, night 18–20°C; 30–55% relative humidity; the natural photoperiod was supplemented with 14 h illumination from Phillips Sun-T Agro 400 Na lights). Bolls were harvested at 25 days after flowering (DAF) in order to obtain immature seeds.

Transgenic flax plants were obtained as described (Renouard et al., 2012). In brief, the *Agrobacterium tumefaciens* strain GV3101 (pGV2260) was used for transformation. The construct contained an 895 bp fragment of the *LuPLR1* gene promoter (accession number AY654626) cloned upstream from the *GUSint* reporter gene (which contains an intron) into the *Hind*III-*Xba*I sites of pGIBin19 plasmid; a transcriptional fusion with the *GUSint* reporter gene was then created.

Transgenic flax plants and wild type plants were grown in the greenhouse of the Centre de Ressources Régionales en Biologie Moléculaire (CRRBM) of the University of Picardie Jules Verne under the conditions mentioned above. Seeds were harvested at different developmental stages for further gene expression studies: S0 (ca 4 DAF, embryo not visible); S1 (ca 10 DAF, embryo 0.5–1 mm); S2 (ca 16 DAF, embryo 2–3 mm); S3 (ca 22 DAF, embryo 4–5 mm, green seed), S4 (ca 28 DAF, embryo 5 mm, seed coat turning brown), S5 (mature seed ca 40 DAF, embryo 5 mm, brown seed coat).

### Purification of SDG

Before chemical and molecular methods were used to elucidate the spatial and temporal distribution of the SDG in flax seed coats, an overall phytochemical reinvestigation was undertaken. In order to optimize analytical procedures and to obtain SDG as a reference material, seeds were separated into seed coats and embryos. Powder obtained by grinding mature seed coats (535 mg) was defatted with *n*-hexane (10 ml, 3 h × 3). The residue was hydrolyzed with 20 ml 20 mM NaOH (50% MeOH and 50% H_2_O solution) overnight in a water bath at 60°c; then the solution was centrifuged at 13,200 rpm (Centrifuge 5415R, Eppendorf, Hamburg, Germany), and the supernatant was evaporated to remove MeOH. The residual aqueous solution was neutralized with 1% (V/V) acetic acid to pH 7.0, and loaded on a Discovery DSC-18 SPE cartridge (10 g, 60 ml. Supelco, Bellefonte, PA, USA), which was conditioned with 20 ml MeOH and then equilibrated with 20 ml H_2_O before using. The eluate of 40 ml H_2_O was discarded and the eluate of 40 ml 75% aqueous MeOH was collected and dried below 40°C in vacuum. The extract was purified on a Merck-Hitachi preparative HPLC system (L-6200A gradient pump, L-4250 UV/Vis detector. Hitachi, Ltd. Tokyo, Japan) using a Purospher RP18e column (5 μm, 250 × 10 mm. Merck KGaA, Darmstadt, Germany). Flow rate 3.5 ml min^-1^; UV detection at 280 nm. The following linear gradient of H_2_O (solvent A) and MeOH (solvent B) was applied: 0 min: 25% B, 35 min: 40% B, 37 min: 95% B, 42 min: 95% B, and 44 min: 25% B, followed by a 5 min equilibration step. SDG (5.4 mg, *R*_t_ 19.1 min) was isolated, and its structure was confirmed by comparing NMR data with those in literature (Chimichi et al., 1999).

### Manual Separation of Mature Seeds

**FIGURE 2:**
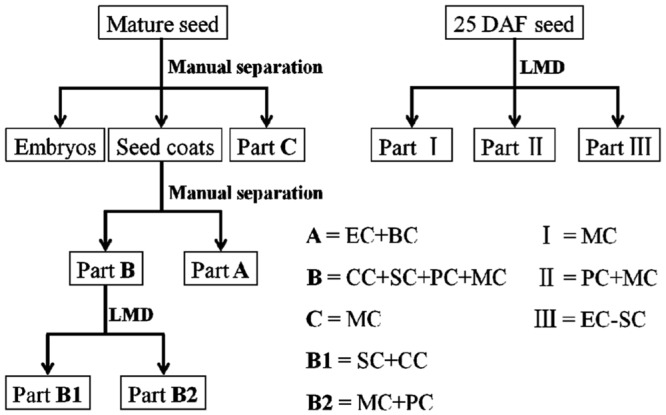
**Schematic representation of workflow involved in separating mature and 25 DAF flax seeds**. Mature flaxseed was divided into embryos and seed coats; seed coats are further separated into two parts, A and B. A contains ECs and BCs, and B consists of CCs, SCs, PCs and MCs. LMD was applied to cut B into two subparts, B1 and B2, which comprise SCs and CCs, PCs and MCs, respectively. Some material was isolated from MCs as part C. After sample preparation, 25 DAF seed coats were directly dissected into three parts, I (MCs), II (MCs + PCs) and III (ECs-SCs). DAF, days after flowering; LMD, laser microdissection; MC, mucilaginous cell; PC, parenchymatous cell; SC, sclerified cell; CC, compressed cell; BC, brown cell; EC, endosperm cell.

The work flow involved in separating mature seeds and 25 DAF seeds is shown in **Figure 2**. Flaxseed was manually separated under a binocular microscope Stemi DV4 (Carl Zeiss MicroImaging GmbH, Jena, Germany). Mature seeds were cut longitudinally around the equator into two halves and embryos were removed from seeds by using a needle. The seed coats contain two parts, outer and inner integuments, which attach to each other loosely (see Supplementary Figure S1A). It was easy to separate the outer and inner integuments by a very fine forceps and a needle under a microscope (see Supplementary Figure S1B). Material of the MC layer can be picked by a needle under the microscope and separated from the rest of the seed coat (see Supplementary Figure S1C).

### Laser Microdissection

The basic work flow of LMD has been reported (Moco et al., 2009). The materials (outer integument for a mature seed or a whole developing seed) were fixed vertically in Jung tissue freezing medium (Leica Microsystems GmbH, Nussloch, Germany), and immediately frozen in liquid nitrogen. Serial cryosections (25 μm thickness) were prepared at -24°C using a cryostat microtome (Leica CM1850, Bensheim, Germany) and directly mounted on PET-Membrane FrameSlides (MicroDissect GmbH, Herborn, Germany).

The laser dissections were performed on the Leica LMD 6000 microdissection system (Leica Microsystems GmbH, Wetzlar, Germany) equipped with a nitrogen solid state diode laser of a short pulse duration (355 nm). The settings were as follows: 20× magnification, laser intensity of 90, laser moving speed of 1 (the slowest). The cut materials were collected in the cap of a 0.5 ml centrifuge tube by gravity. The pictures were taken by an integrated camera HV-D20P (Hitachi, Tokyo, Japan).

### Alkaline Hydrolysis of Separated Samples

Manually separated samples were transferred into 20 ml glass vials. Then 4 ml 20 mM NaOH (in MeOH/H_2_O 1:1) was added. After magnetic stirring at ambient temperature for 3 h, the solutions were neutralized with 1% (V/V) acetic acid. Laser-microdissected samples were transferred into HPLC vials (1.5 ml), 1 ml 20 mM NaOH (in MeOH/H_2_O 1:1) was added and the samples were hydrolyzed correspondingly in a water bath at 60°C. The hydrolysis solutions were dried in vacuum < 40°C.

### NMR Analysis of Alkaline Hydrolyzed Samples

The manually separated samples were extracted with 600 μl MeOH-*d_4_* (99.96%, Deutero GmbH, Kastellaun, Germany) in an ultrasonic bath for 2 min and filtered into 5 mm diameter NMR tubes. The LMD samples were prepared in the same way but with 90 μl solvent and 2 mm NMR tubes. ^1^H NMR and 2D spectra (^1^H,^1^H COSY and HSQC) were recorded at 300 K in a Bruker Avance 500 NMR spectrometer equipped with a 5 mm cryogenic TCI probe (Bruker-Biospin, Rheinstetten, Germany). ^1^H NMR spectra were recorded with 1024 scans. The residual HDO signal was suppressed using the PURGE sequence (Simpson and Brown, 2005). The residual signals of MeOH-*d_4_* at δ_1H_ 3.31 and δ_13C_ 49.00 were used as chemical shift references (Gottlieb et al., 1997).

### HPLC Analysis of Alkaline Hydrolyzed Samples

Analytical HPLC was performed on an Agilent series HP1100 (binary pump G1312A, autosampler G1367A, diode array detector (DAD) G1315A, 200–700 nm) (Agilent Technologies, Waldbronn, Germany). A LiChrospher RP18 column (5 μm, 250 × 4 mm. Merck KGaA, Darmstadt, Germany) was used with a linear binary gradient of H_2_O (solvent A) and MeCN (solvent B), both containing 0.1% (V/V) trifluoroacetic acid, with a flow rate of 0.8 ml min^-1^ at 25°C as follows: 0 min: 5% B, 35 min: 25% B, 37 min: 95% B, 47 min: 95% B, 50 min: 5% B, and 60 min: 5% B. The injection volume was 5 μl. The HPLC eluate was monitored by DAD at 280 nm.

### Lignin Analysis

Entire integuments of mature flaxseed were ground (grinder MM301, Retsch, Germany) and ultrasonicated for 10 min in MeOH / H_2_O 1:1 to extract the lignan macromolecule. The residue was then submitted to thioacidolysis. Another sample was submitted to thioacidolysis without prior extraction. The monomer composition of the labile ether lignin fraction was determined by thioacidolysis, which specifically disrupts the non-condensed intermonomer linkages (alkyl-aryl ether). The reaction was performed using 10 mg teguments and ethanethiol/BF_3_ etherate/dioxane reagent as detailed previously (Lapierre et al., 1986). Tetracosane was added as an internal standard. After 4 h, the mixture was extracted with CH_2_Cl_2_ (3 × 25 ml). Guaiacyl (G) and syringyl (S) thioethylated monomers were determined as their trimethylsilyl derivatives using a gas chromatograph equipped with a fused silica capillary DB1 column (30 m × 0.3 mm) (J&W Scientific, Folsom, CA, USA) and flame ionization detector. The temperature gradient was 160–280°C at 2°C min^-1^.

### Manual Dissection of Developing Seeds for Gene Expression Studies

For *GUS* activity assays, immature seeds of stages S2, S3, and S4 were cut longitudinally with a scalpel and the embryos were removed with a needle. S0 and S1 early stages (embryo too small) and S5 (mature seeds) were used as a whole. For RT-PCR experiments, S3 developing seeds (wild type) were cut longitudinally on one side with a scalpel; the embryos were removed with a needle, and inner and outer integuments were separated under binocular microscope using fine forceps.

### Quantitative GUS Activity Assay

ß-Glucuronidase activity of immature seeds (whole seeds, manually separated seed coats and embryos) was estimated as described by Renouard et al. (2012) using 4-methylumbelliferyl-β-D-glucuronide (4-MUG, Sigma) as substrate. Immature seeds of stages S2, S3, and S4 were cut longitudinally with a scalpel and the embryos were removed with a needle, GUS activities of seed coats and embryo were measured separately. Whole seeds were used for earliest stages (S0 and S1, embryo too small), and latest stage S5 (mature seeds).

### Histochemical GUS Assays

Seeds (harvested from transgenic plants grown in the greenhouse) at different stages of development (S1, S2, S3, and S5) were cut transversally and subjected to histochemical staining for *GUS* activity as described by Jefferson et al. (1987) and modified (Kosugi et al., 1990) to avoid background that could be due to non-specific endogenous *GUS* activity (i.e., with 20% MeOH in 5-bromo-4-chloro-3-indolyl-β-D-glucuronic acid (X-Gluc) solution). A K^+^ ferricyanide/ferrocyanide mixture (2 mM of each) was also added to the incubation buffer to prevent the diffusion of the indoxyl derivative before its oxidative dimerization.

Semi-thin sections were then obtained as described by Hawkins et al. (2002). Briefly, *GUS*-stained samples were fixed in formalin acetic alcohol (FAA) [10% formalin (37% formaldehyde stabilized with MeOH), 5% glacial acetic acid, 60% EtOH] for 24 h, dehydrated and embedded in paraffin. Semi-thin sections (5 μm) were made on a RM 2145 rotary microtome (Leica, Wetzlar, Germany), then deparaffinized and counter-stained with periodic acid Schiff to visualize the cell walls in each tissue before permanent mounting and microscopic observation. Pictures were taken with a Nikon Coolpix 5400 digital camera (Nikon, Tokyo, Japan). The same experiments were conducted on different stage wild type seeds as negative control.

### RNA Extraction and RT-PCR of the Transcript

RNA was isolated from the embryo, inner integument and outer integument of S3 (ca 22 DAF) immature seeds (separated using scalpel and forceps under binocular microscope) using the protocol described (Gutierrez et al., 2006). S3 developing seeds (wild type) were cut longitudinally on one side with a scalpel; the embryos were removed with a needle, and inner and outer integuments were separated under binocular microscope using fine forceps.

RT-PCR detection of the LuPLR1 transcript was performed as described by Renouard et al. (2012). A 939 bp fragment of the *LuPLR1* cDNA was amplified using *PLRF1* forward primer (5′-ATGGGGCGGTGCAGAGTTCT-3′) and *PLR-R1* reverse primer (5′-TCAAAGGTAGATCATCAGA-3′) designed from the flax *LuPLR1* cDNA sequence (accession number AX191955). A PCR product (632 bp) corresponding to the exon 2 of the *ACTIN* gene was amplified with ACT-F2 forward primer (5′-TCTGGAGATGGTGTGAGCCACAC-3′) and ACT-R2 reverse primer (5′-GGAAGGTACTGAGGGAGGCCAAG-3′) designed from the tobacco sequence. cDNA fragments were amplified during 25, 27, and 30 cycles.

## Results

### Manual Separation and Laser Microdissection of the Seed Coat Cell Layers

**FIGURE 3:**
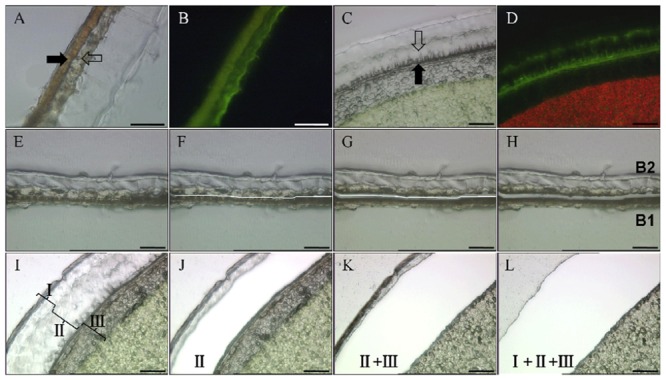
**Anatomical pictures and workflow of laser microdissection (LMD) taken with a HV-D20P camera integrated in the LMD 6000 micro dissection system. (A,B)** Micrographs of part B of mature flaxseed coats under light and fluorescence, respectively. The full arrow shows SCs, and the empty arrow shows PCs. Bars represent 100 μm. **(C,D)** Anatomical structures of 25 DAF seed coats under light and fluorescence. Developing SCs and PCs are marked by full and empty arrows, respectively. Bars represent 50 μm. The fluorescence optic settings are as follows: excitation = 450–490 nm, dicroic mirror = 510 nm, and emission = 515 nm. **(E–H)** The LMD work flow of dividing part B is as follows: **(E)** Tissue of part B before laser cutting, **(F)** drawing a cutting line using the software of the laser microscope, **(G)** laser cutting alongside the software-drawn line, and **(H)** separated subparts B1 and B2 after cutting. Bars represent 100 μm. **(I–L)** Work flow of separating 25 DAF seed coats into three parts I to III. **(I)** Tissue of immature seed coats before laser cutting, **(J)** tissue after cutting part II, **(K)** tissue after successively cutting parts II and I, **(L)** tissue after successively cutting parts II, I and III. Bars represent 50 μm. DAF, days after flowering; PC, parenchymatous cell; SC, sclerified cell.

First, the embryo was removed from the mature seed and embryo extracts were analyzed by HPLC and NMR, demonstrating the absence of SDG (data not shown). Then the seed coats were manually separated into two parts (see Supplementary Figure S1B), A (inner integument, 10.5 mg) and B (outer integument, 13.0 mg). Part A contains layers of ECs and BCs, and part B comprises four layers, from inner to outer: CCs, SCs, PCs and MCs (**Figures 1B,C, 2** and **3A,B**). Material from MCs (1.8 mg) was manually collected from seed coats as part C (see Supplementary Figure S1C). Part B obtained by manual separation was cryosectioned. LMD was used to dissect cell layers of part B into two subparts B1 (CCs + SCs, 380 μg) and B2 (PCs + MCs, 440 μg) (**Figures 3E–H**). The material includes the supporting polyethylene terephthalate (PET) membrane of the frame slide, which was unavoidably cut together with the cells. LMD turned out to be excellent for dissecting seed coats because of the rigid architecture of their cell walls and relatively low water content. The characteristic cell shape and the autofluorescence of seed coat cells were used to identify the target material without staining.

Immature seeds were harvested at 25 DAF. At that developmental stage, the ultrastructure of seed coats (**Figures 3C,D**) is not yet fully developed and manually separating cell layers is very difficult. Hence, the entire seed was subjected to cryosectioning. LMD was used to dissect seed coats into three different parts: I (MCs, 200 μg), II (PCs + MCs, 250 μg) and III (from SCs to ECs, 570 μg) (**Figures 3I–L**). Again, the collected masses contain a portion of the PET slide membrane. The material sampled by LMD was subjected to HPLC and NMR analyses. Some blank membrane pieces with fixation medium was collected and analyzed as control. No clear signals in NMR spectra and peaks in HPLC chromatograms were observed.

### NMR Analysis Results of Alkaline Hydrolyzed Samples

**FIGURE 4:**
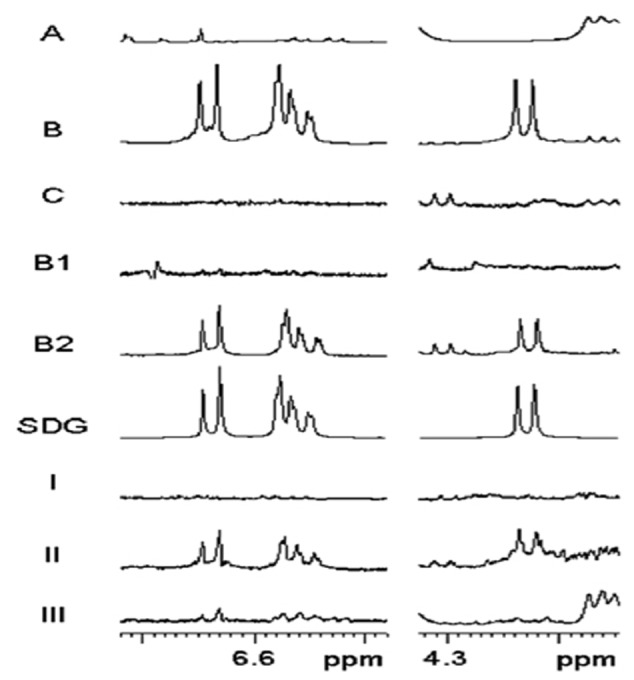
**^1^H NMR spectra (500 MHz, MeOH-*d_4_*) of hydrolyzed material from different cell types of flaxseed coats.** ^1^H NMR spectra (A,B,C,B1,B2) were obtained from mature seed samples. (SDG) ^1^H NMR spectrum of secoisolariciresinol diglucoside. ^1^H NMR spectra (I,II,III) were obtained from 25 DAF seed samples. For details of sample preparation, see **Figures 1** and **2**. Partial ^1^H NMR spectra show characteristic SDG signals of the aromatic protons (H-5/5′: δ6.64; H-2/2′: 6.58; H-6/6′: 6.56) and the doublet of the proton at the anomeric center of glucose (H-1″/1‴: δ 4.23). DAF, days after flowering.

Secoisolariciresinol diglucoside was released from the separated samples by alkaline hydrolysis. In the ^1^H NMR spectra (**Figure 4**), SDG is readily determined by the diagnostic SDG signals of the trisubstituted phenyl ring (H-5/5′: δ6.64; H-2/2′: 6.58; H-6/6′: 6.56) and the doublet of the proton at the anomeric center of glucose (H-1″/1‴: δ 4.23). Intense signals of SDG appeared in the NMR spectra obtained from extracts of the outer integument (B) of the mature seed coats. However, SDG can hardly be observed in part A (ECs + BCs) and **C** (MCs). In LMD samples, SDG was the major component of B2 (MCs + PCs), and SDG was not found in B1 (CCs + SCs). In the immature seeds (25 DAF), SDG was detected mostly in part II, which corresponds to MCs + PCs. A trace of SDG was found in part III (from SCs to ECs), and no SDG was observed in part I (MCs).

### HPLC Analysis Results of Alkaline Hydrolyzed Samples

Secoisolariciresinol diglucoside concentrations in seed coat samples were quantified using HPLC-DAD. The chromatograms (280 nm) of extracts from cells of mature seeds and 25 DAF seeds are shown in Supplementary Figure S2, and the concentration values, which were calculated by using an SDG linearity equation, are listed in **Table 1**. The HPLC and NMR data are consistent. In mature seed coats, part B contains 3.1% (w/w) SDG, and the concentrations of SDG in parts A and C were clearly below 0.1 % (calculated as 0.01% in A and 0.06% in C). SDG was detected only in B2 in a concentration of 3.1%. In 25 DAF seed coats, SDG was mostly found in part II, most of whose material was from developing PCs.

**Table 1:** Secoisolariciresinol diglucoside (SDG) contents of seed coat cell layers determined by HPLC after alkaline hydrolysis.

		Part	Cell layer	Separated material amount (mg)	Peak area (mAU)	SDG amount (μg)	SDG concentration (%)
Mature seeds	Manual separation	A	EC + BC	10.50	3.64	1.6	<<0.1
		B	CC + SC + PC + MC	13.00	1418.22	400.0	3.1
		C	MC	1.80	1.69	1.0	<0.1
	LMD	B1	CC + SC	0.38	ND		
		B2	PC + MC	0.44	46.11	13.5	3.1
25 DAF seed	LMD	I	MC	0.20	0.74	0.7	0.4
		II	PC + MC	0.25	24.00	7.3	2.9
		III	EC-SC	0.57	2.98	1.4	0.2

*SDG concentrations were calculated as the percent of SDG amounts in separated material amounts. ND means no detectable peak in chromatogram.*

### Quantification of *LuPLR1* Gene Expression by β-Glucuronidase (*GUS*) Activity Assay

**FIGURE 5:**
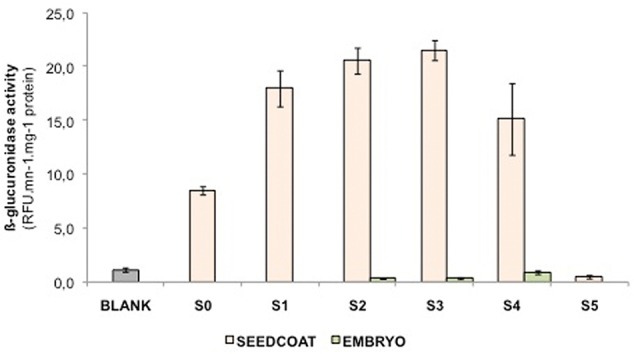
***GUS* activities from embryo and seed coats in the developing seeds of flax plants stably transformed with an 895-bp *LuPLR1* promoter-*GUS* reporter gene construct are shown.** S0, S1, S2, S3, S4, and S5 correspond to ca 4, 10, 16, 22, 28, and 40 days after flowering, respectively, and WT represents the activity measured in an S3 wild type whole seed.

*LuPLR1* promoter transcriptional activity was estimated by measuring *GUS* activity in seeds of transgenic plants (*LuPLR1* promoter-*GUS* reporter gene construct) (**Figure 5**). Enzyme activity was detectable as early as S0, then increased during phases of seed development to reach a maximum at S3 and finally decreased during the seed maturation phase (S4, S5). Manual separation at stages S2, S3, and S4 revealed that activity was mainly localized in seed coats.

### Cell-Specific *LuPLR1* Gene Expression by Histochemical *GUS* Assay

**FIGURE 6:**
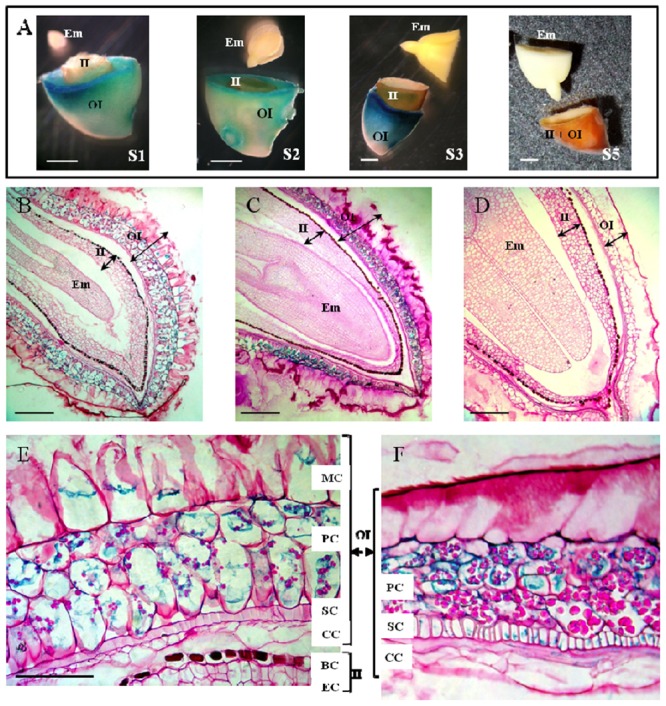
**Histochemical studies of the localization of *LuPLR1* promoter-driven *GUS* gene expression in transgenic flaxseed at different developing stages. (A)** *GUS* assays performed on transversely cut seeds at S1, S2, S3 and mature seeds. Bars represent 1 mm. **(B–D)** Semi-thin sections (5 μm) of S2 (**B**, longitudinal), S3 (**C**, longitudinal) and S5 (**D**, transverse) seeds assayed by *GUS* activities. Bars represent 250 μm. **(E,F)** *GUS* staining in semi-thin sections (5 μm) of S2 **(E)** and S3 **(F)** seed coats. Bars represent 50 μm. OI, outer integument (Part B); II, inner integument (Part A); Em, embryo. MC, mucilaginous cell; PC, parenchymatous cell; SC, sclerified cell; CC, compressed cell; BC, brown cell; EC, endosperm cell.

Histochemical *GUS* assay performed on developing seeds at stages S1, S2, S3 and mature seeds revealed strong *GUS* activities localized in the outer integument (Part B) of S1, S2, and S3 but not in mature seeds. No enzyme activity could be detected in the inner integument (Part A) and the embryo regardless of the developmental stage (**Figures 6A–D**).

Semi-thin sections of seeds preliminary assayed for *GUS* activity allowed more precise localization of *LuPLR1* promoter transcriptional activity. *GUS* activity was mainly localized in the PCs, no matter what developmental stage was studied (S1, data not shown; S2 and S3) except for the mature seeds, in which no staining could be detected (**Figures 6B–F**). Weak *GUS* activity could be detected in the MCs of S2 developing seeds. Staining was also observable in SCs and in CCs from S3 seed coats. No *GUS* activity was observed in BCs and ECs regardless of the development stage considered. In the seeds of wild type plants, no *GUS* staining could be observed in any cell layer and at any developmental stage studied (data not shown).

### RT-PCR Detection of *LuPLR1* Transcript in Wild Type Plants

**FIGURE 7:**
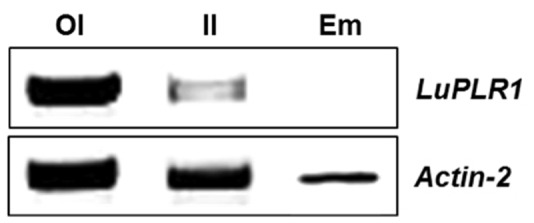
***LuPLR1* gene expression in the outer integument (OI), inner integument (II) and embryo (Em) of S3 (ca 22 days after flowering) wide type developing seeds analyzed by RT-PCR.** Total RNA isolated from manually separated tissues was subjected to RT-PCR semi-quantitative analysis using an *ACTIN* gene as internal control. 27-cycle PCR products (10 μm) were loaded on 1 % (w/v) agarose gel. OI, outer integument (Part B); II, inner integument (Part A); Em, embryo.

Since gene expression was highest in S3 (ca 22 DAF) of transgenic seeds and then dropped, this developmental stage was used to detect *LuPLR1* transcript by RT-PCR in manually separated outer integument (Part B), inner integument (Part A) and embryo. *LuPLR1* transcript was mainly detected in the outer integument of transgenic seeds. The results shown in **Figure 7** indicate that *LuPLR1* is more expressed in the outer integument as its transcript was intensely detected in this tissue. Only a weak *LuPLR1* signal was detected in the inner integument, while no transcripts could be detected in the embryo, confirming the results of quantitative *GUS* assays.

### Lignin Analysis

Chemical characterization and quantification of lignin were performed on material from mature seed coats. Thioacidolysis was used as previously described for flax stem material (Day A. et al., 2005). Before and after extraction with MeOH, no syringyl lignin was found; only guaiacyl lignin was quantified (non-extracted sample: 22.8 ± 4 μmol g^-1^ sample; MeOH-extracted sample 15.3 ± 1 μmol g^-1^ sample). Although these values are low, they indicate the occurrence of lignin or lignin-like structures in the flax seed coat. The tri-substituted aromatic rings of the guaiacyl lignin are identical with those of the SDG.

## Discussion

### SDG Localization in Mature and Immature Flaxseed

The cell layer-specific localization of SDG in flaxseed coats and the temporal progression of SDG production during maturation have been investigated by chemically analyzing microdissected samples and gene expression. As previously reported (Hano et al., 2006; Attoumbré et al., 2010, 2011), and also indicated by our NMR and HPLC analyses, SDG accumulates in seed coats, not in embryos.

Based on the NMR and HPLC analyses of samples from mature seed coats collected by manual separation, a high concentration of SDG (3.1%) was located in the outer integument (part B) but only a trace concentration (0.06%) was found in the layer of mucilagineous cells (MCs, part C). Because the constituents of MCs are pectins (Naran et al., 2008), and no phenolics have been found there before, it seems reasonable to assume that the trace of SDG detected in MCs resulted from contamination by material from the adjacent PC layer displaced during manual separation. In the two subparts divided from B by LMD, SDG was detected only in B2, which contains MCs and PCs, but not in B1, which contains CCs and SCs (**Figure 4**, **Table 1**; Supplementary Figure S2). The concentration of SDG in the inner integument (part A), which was as low as 0.01%, was probably due to contamination, again originating from PCs during manual separation. Therefore, according to chemical analysis, all SDG in mature seeds was thought to locate in the PC layer of the outer integument.

The NMR and HPLC results (**Figure 4**, **Table 1**; Supplementary Figure S2) obtained from 25 DAF seeds indicated that the highest level of SDG was found in part II of the seed coats and minor levels were found in parts III and I of the seed coats. Thus, the PC layer is the major location of SDG in developing seeds already; around 78% of the total amount of SDG in 25 DAF seeds was detected in this cell layer. Unlike mature seeds, seeds in the early developing stage do not contain a complete PC layer. The PC layer, which is around 80 μm wide measured in the middle of 25 DAF seeds, is filled with liquid-like material. This could explain how a small amount of SDG is found in part I, because the material in PCs may easily delocalize during cryosectioning or the preparation of the slides, and LMD. Material diffusion may also cause the boundary between SCs and PCs to become indistinct, another reason may be SDG detected in part III of 25 DAF seeds.

This detection of high levels of SDG in PCs is slightly contradictory with a previous study performed in our lab (BIOPI). An innovative immunolocalization approach was developed to detect SDG in flaxseed (Attoumbré et al., 2010) and tended to show that lignans were mainly accumulated in the secondary cell walls of SCs. As suggested by Dalisay et al. (2015), it is likely that the reported immunoreaction is not very specific to lignans including the SDG-HMG oligomer complex. In fact, this study used polyclonal antibodies which were developed using secoisolariciresinol (which occurs in flaxseed coats in very low levels only), not SDG nor the SDG-HMG complex, as a hapten for immunization. Moreover, considering that cross-reactivity for SDG was 22.1% and for other lignans up to 32.8%, the specificity of the antibodies to SDG used was relatively low. As pointed out in Attoumbré et al. (2010, 2011) the specificity of the antibodies is relative to the substituent at the aromatic ring and at C-8 /C-8′ of the lignan side chains. Therefore, the ester-type bond, which connects SDG through HMGA to SDG-HMG oligomer complexes (Ford et al., 2001), may affect the interaction with the antibodies and monolignols (coniferyl alcohol, *p*-coumaryl alcohol) may cross-react with the antibodies used (Attoumbré et al., 2010, 2011). To confirm this hypothesis, the use of specific antibodies versus lignin would be very helpful, notably the use of the KM2 antibodies specific of 8-8′ linkage developped in Kiyoto et al. (2013).

Flax lignin has been reported as highly condensed and rich in guaiacyl moieties (G lignin) (Day A. et al., 2005) and our data indicate the occurrence of G lignin or lignin-like structures in the flax seed coat. Our finding that flax seed coats contain guaiacyl units but no syringyl units in the lignin supports the suggestion that structures other than SDGs could be responsible for the positive reaction of antibodies raised against SDG reported (Attoumbré et al., 2010). Another explanation for the positive immunolabelling in SCs (Attoumbré et al., 2010) could be that the *PLR* product (aglycone SECO) might have been synthesized but not converted to a diglucosylated and complexed form (SDG-HMG); from this form, it could be incorporated in the cell wall in a post-lignification “infusion” process.

Another point to be mentioned is the poor solubility of SDG and SDG oligomers in water (Zhang et al., 2009) which may result in leakage from PCs to their neighbor cells, i.e., SCs during the ethanol dehydration steps applied by Attoumbré et al. (2010); meanwhile, SDG may stick to the lignified SC walls as these are more hydrophobic than the walls of PCs.

### Location of SDG Biosynthesis

Two enzymatic assays (quantitative and histochemical) combined with semi-quantitative RT-PCR measurements confirmed that *LuPLR1* is expressed mainly in the seed coats. *LuPLR1* is responsible for the synthesis of the major (+)-secoisolariciresinol enantiomer (87%) while a minor quantity of the (–)-secoisolariciresinol enantiomer (13%) is synthesized by *LuPLR2* (Hemmati et al., 2010). The very weak β-glucuronidase (*GUS*) activity detected in embryos has to be attributed to non-specific *GUS* that has already been reported in seeds of a number of plants (Hu et al., 1990; Hänsch et al., 1995). *GUS* activities measured at different developmental stages in the coats and embryos of transgenic flax seeds containing the *LuPLR1* gene promoter were in accordance with those of previously published semi-quantitative RT-PCR experiments (Hano et al., 2006), confirming that the SDG biosynthetic pathway is active already at S0 and reaches a maximum during S1, S2, and S3 (**Figure 5**). RT-PCR employed for the detection of the *LuPLR1* transcript in three separated parts (embryos, inner and outer integuments) of S3 seeds from wild type plants indicated that *LuPLR1* transcript is mainly detected in the outer integument, which also has been observed in transgenic plants. The weak amplification signal in the inner integument could be attributed to tissue contamination during manual dissection, confirming data of NMR and HPLC analyses. Co-localization of SDG and *LuPLR1* expression suggests that glucosylation of (+)-secoisolariciresinol (**4** in **Figure 1A**) also occurs in the PCs, which both synthesize (+)-secoisolariciresinol and accumulate SDG.

Generally, data on histolocalization of *LuPLR1* promoter-driven *GUS* expression at development stages S1 – S3 show that PCs are the major location of SDG synthesis, which LMD / HPLC and NMR data on SDG accumulation in mature and 25 DAF flaxseed also show. Histochemical assay did not detect *GUS* in mature seeds. This is consistent with data showing that *GUS* activity is very weak in S5 flaxseed, suggesting that SDG synthesis is completed before maturity.

*GUS* activity observed in some MCs at S2, and in some SCs and CCs at S3, suggesting that SDG in layers of non-PCs is due to the delocalization of PC material during cryosectioning and mounting. Otherwise, *GUS* would be detected in all MCs, SCs or CCs. The literature offers several other possible explanations of visible *GUS* staining in partial SCs and CCs at S3 of seed development as the result of *LuPLR1* promoter transcriptional activity. Expression of *LuPLR1* in SCs could be related to the fact that these cells are undergoing lignification. It has been proven using immunolocalization and *in situ* hybridization techniques in *Forsythia intermedia*, that lignan biosynthetic genes such as dirigent proteins and *PLR* genes are expressed in xylem and other lignifying tissues (Burlat et al., 2001; Kwon et al., 2001). In flax, stem cells display a contrasting cell wall structure with a lignified inner tissue and a hypolignified outer stem including bast fiber which accumulate substantial amount of (neo)lignan glucosides. This differential allocation of (neo)lignans and lignin into these tissues was associated with different gene expression patterns and interestingly three *PLR-like* genes were associated with the progressive formation of xylem tissue and extensive lignification of the cell wall (Huis et al., 2012). In Arabidopsis, *AtPRR1*, a *LuPLR1* ortholog encoding for a pinoresinol reductase, is co-expressed with genes involve in secondary cell wall biosynthesis and its gene promoter activity was increased by SND1 and MYB46, two important master regulators of secondary cell wall formation (Zhao et al., 2015). Moreover it has been demonstrated that the overexpression of two *Pinus taeda* MYB transcription factors (PtMYB1 and PtMYB8), controlling the gene expression of monolignol biosynthetic genes, results in the upregulation of two *PLR-like* encoding genes in *Picea glauca* (Bomal et al., 2008). Lignans such as pinoresinol can also be found in a methanol-soluble oligolignol fraction of lignifying xylem (Morreel et al., 2004) or in differentiating tracheary elements (Tokunaga et al., 2005) and are therefore able to be incorporated into the lignin polymer. In some Pinaceae species, lignans (8-8′ linked) and neo-lignans (8-5′ linked) are known to be infused in the heartwood after lignification, increasing its resistance and durability (Gang et al., 1999). The hydrophilic knotwood extracts of different coniferous species have been also shown to contain mainly lignans, among which lariciresinol, secoisolariciresinol or matairesinol can be found in variable amounts according to the species (Willför et al., 2003).

### Applications of LMD in Plant Metabolite Studies

The conventional histological methods to study the spatial distribution of metabolites in microscopic plant samples make use of *in situ* labeling and (or) staining techniques. Due to the structural analogy among metabolites of many plants, the specificity of histological method is relative low. Recent developments in mass spectrometry (Svatoš, 2011) and Raman imaging (Freudiger et al., 2008) are promising tool in metabolic profiling. However, these techniques mostly focus on metabolites on sample surfaces and do not allow detection of three-dimensional distribution of metabolites within the tissue. NMR, though representing the most informative analytical method and being able to provide data on three-dimensional spatial distribution, is of limited suitability to identify metabolites directly from plant samples, due to its moderate sensitivity. In order to take advantage of its superior properties for metabolic profiling, NMR has to be combined with appropriate sampling methods. LMD was used for sampling plant material for DNA, RNA and protein analyses (Kehr, 2001, 2003; Day R.C. et al., 2005; Nelson et al., 2006, 2008) and to dissect plant material for the analysis of both primary metabolites (Angeles et al., 2006; Obel et al., 2009; Thiel et al., 2009; Schiebold et al., 2011) and secondary metabolites (Kajiyama et al., 2006; Li et al., 2007; Schneider and Hölscher, 2007; Hölscher et al., 2009; Abbott et al., 2010). As presented here, LMD combined to NMR and HPLC, allows for the quantitative spatio-temporal analysis of metabolites in plant cells. Future advances in LMD, such as improvement in target cell-recognizing software, multi-target sampling, hyphenation with sensitive detectors, together with NMR sensitivity enhancement, will enable more efficient cell-specific identification and quantification of metabolites in plant tissue.

## Conclusion

Laser microdissection was applied for the first time in combination with chemical treatment, and two analytical methods, NMR and HPLC, to elaborate the spatio-temporal location of metabolites in plants on the cellular level. This work also shows that LMD is able to provide material sufficient for multiple analytical manipulations. The study constitutes a technical advance for the better understanding of the regulation of SDG biosynthetic pathway in flaxseed. Not only it allowed cell layer-specific location and time course analysis of SDG synthesis but also could be a powerful tool for future transcriptomic studies (Hölscher and Schneider, 2008; Nelson et al., 2008; Matas et al., 2011; Olofsson et al., 2012), the isolation of transcription factors controlling SDG synthesis in the PC layer being a great challenge.

The ecological role of flax lignans in the developing and mature seed still awaits to be identified. However, the location of the SDG polymer complex in the parenchymatic cells just below the external MC layer suggests chemical defense against insects and microorganisms. An additional physiological function of lignans in seed development or germination is possible. It is also plausible that the SCs, supplementary to their role in providing the flaxseed scaffold, represent a mechanical defense barrier against invading organisms. Further studies are required in order to reveal the benefit to the flax plant of producing and accumulating lignans in the seed coat.

## Author Contributions

FM and BS contributed substantially to the conception of the work. JF and AR prepared the samples and performed the laser microdissection experiments; they also performed the NMR and HPLC experiments. JF, AR, FM, and BS analyzed these obtained data. SR, CH, and FL were in charge of all the aspects of molecular biology. BC performed the lignin analysis. All the authors contributed in drafting the manuscript or in revising it critically for important intellectual content and approved the final version to be published. The authors agree to be accountable for all aspects of the work in ensuring that questions related to the accuracy or integrity of any part of the work are appropriately investigated and resolved.

## Conflict of Interest Statement

The authors declare that the research was conducted in the absence of any commercial or financial relationships that could be construed as a potential conflict of interest.

## References

[B1] Abbott E. Hall D. Hamberger B. Bohlmann J. (2010). Laser microdissection of conifer stem tissues: isolation and analysis of high quality RNA, terpene synthase enzyme activity and terpenoid metabolites from resin ducts and cambial zone tissue of white spruce (*Picea glauca*). *BMC Plant Biol.* 10:106 10.1186/1471-2229-10-106 PMC3095273 20540781

[B2] Adolphe J. L. Whiting S. J. Juurlink B. H. J. Thorpe L. U. Alcorn J. (2010). Health effects with consumption of the flax lignan secoisolariciresinol diglucoside. *Br. J. Nutr.* 103 929–938. 10.1017/S0007114509992753 20003621

[B3] Angeles G. Berrio-Sierra J. Joseleau J. P. Lorimier P. Lefèbvre A. Ruel K. (2006). Preparative laser capture microdissection and single-pot cell wall material preparation: a novel method for tissue-specific analysis. *Planta* 224 228–232. 10.1007/s00425-006-0285-1 16721624

[B4] Attoumbré J. Bienaimé C. Dubois F. Fliniaux M. A. Chabbert B. Baltora-Rosset S. (2010). Development of antibodies against secoisolanciresinol – Application to the immunolocalization of lignans in *Linum usitatissimum* seeds. *Phytochemistry* 71 1979–1987. 10.1016/j.phytochem.2010.09.002 20888604

[B5] Attoumbré J. Laoualy A. B. M. Bienaimé C. Dubois F. Baltora-Rosset S. (2011). Investigation of lignan accumulation in developing *Linum usitatissimum* seeds by immunolocalization and HPLC. *Phytochem. Lett.* 4 194–198. 10.1016/j.phytol.2011.03.004

[B6] Axelson M. Sjövall J. Gustafsson B. E. Setchell K. D. R. (1982). Origin of lignans in mammals and identification of a precursor from plants. *Nature* 298 659–660. 10.1038/298659a0 6285206

[B7] Bomal C. Bedon F. Caron S. Mansfield S. D. Levasseur C. Cooke J. E. K. (2008). Involvement of *Pinus taeda* MYB1 and MYB8 in phenylpropanoid metabolism and secondary cell wall biogenesis: a comparative in planta analysis. *J. Exp. Bot.* 59 3925–3939. 10.1093/jxb/ern234 18805909 PMC2576632

[B8] Burlat V. Kwon M. Davin L. B. Lewis N. G. (2001). Dirigent proteins and dirigent sites in lignifying tissues. *Phytochemistry* 57 883–897. 10.1016/S0031-9422(01)00117-0 11423139

[B9] Chimichi S. Bambagiotti-Alberti M. Coran S. A. Giannellini V. Biddau B. (1999). Complete assignment of the ^1^H and ^13^C NMR spectra of secoisolariciresinol diglucoside, a mammalian lignan precursor isolated from *Linum usitatissimum*. *Magn. Reson. Chem.* 37 860–863. 10.1002/(SICI)1097-458X(199911)37:11<860::AID-MRC559>3.0.CO;2-A

[B10] Dalisay D. S. Kim K. W. Lee C. Yang H. Rubel O. Bowen P. B. (2015). Dirigent protein-mediated lignan and cyanogenic glucoside formation in flax seed: integrated omics and MALDI mass spectrometry imaging. *J. Nat. Prod.* 78 1231–1242. 10.1021/acs.jnatprod.5b00023 25981198

[B11] Day A. Ruel K. Neutelings G. Crônier D. David H. Hawkins S. (2005). Lignification in the flax stem: evidence for an unusual lignin in bast fibers. *Planta* 222 234–245. 10.1007/s00425-005-1537-1 15968509

[B12] Day R. C. Grossniklaus U. Macknight R. C. (2005). Be more specific! Laser-assisted microdissection of plant cells. *Trends Plant Sci.* 10 397–406. 10.1016/j.tplants.2005.06.006 16027030

[B13] Ford J. D. Huang K. S. Wang H. B. Davin L. B. Lewis N. G. (2001). Biosynthetic pathway to the cancer chemopreventive secoisolariciresinol diglucoside-hydroxymethyl glutaryl ester-linked lignan oligomers in flax (*Linum usitatissimum*) seed. *J. Nat. Prod.* 64 1388–1397. 10.1021/np010367x 11720519

[B14] Freudiger C. W. Min W. Saar B. G. Lu S. Holtom G. R. He C. W. (2008). Label-free biomedical imaging with high sensitivity by stimulated Raman scattering microscopy. *Science* 322 1857–1861. 10.1126/science.1165758 19095943 PMC3576036

[B15] Gang D. R. Kasahara H. Xia Z. Q. Vander Mijnsbrugge K. Bauw G. Boerjan W. (1999). Evolution of plant defense mechanisms – Relationships of phenylcoumaran benzylic ether reductases to pinoresinol-lariciresinol and isoflavone reductases. *J. Biol. Chem.* 274 7516–7527. 10.1074/jbc.274.11.7516 10066819

[B16] Ghose K. Selvaraj K. McCallum J. Kirby C. W. Sweeney-Nixon M. Cloutier S. J. (2014). Identification and functional characterization of a flax UDP-glycosyltransferase glucosylating secoisolariciresinol (SECO) into secoisolariciresinol monoglucoside (SMG) and diglucoside (SDG). *BMC Plant Biol.* 14:82 10.1186/1471-2229-14-82 PMC3986616 24678929

[B17] Gottlieb H. E. Kotlyar V. Nudelman A. (1997). NMR chemical shifts of common laboratory solvents as trace impurities. *J. Organ. Chem.* 62 7512–7515. 10.1021/jo971176v 11671879

[B18] Gutierrez L. Conejero G. Castelain M. Guénin S. Verdeil J. L. Thomasset B. (2006). Identification of new gene expression regulators specifically expressed during plant seed maturation. *J. Exp. Bot.* 57 1919–1932. 10.1093/jxb/erj138 16606634

[B19] Hano C. Martin I. Fliniaux O. Legrand B. Gutierrez L. Arroo R. R. J. (2006). Pinoresinol-lariciresinol reductase gene expression and secoisolariciresinol diglucoside accumulation in developing flax (*Linum usitatissimum*) seeds. *Planta* 224 1291–1301. 10.1007/s00425-006-0308-y 16794840

[B20] Hänsch R. Koprek T. Mendel R. R. Schulze J. (1995). An improved protocol for eliminating endogenous β-glucuronidase background in barley. *Plant Sci.* 105 63–69. 10.1016/0168-9452(94)04033-D

[B21] Hawkins S. Pilate G. Duverger E. Boudet A. M. Grima-Pettenati J. (2002). “The use of GUS histochemistry to visualise lignification gene expression during wood formation,” in *Wood Formation in Trees – Cell and Molecular Biology Techniques* ed. Chaffey N. (London: Taylor & Francis Inc) 271–295.

[B22] Hemmati S. von Heimendahl C. B. I. Klaes M. Alfermann A. W. Schmidt T. J. Fuss E. (2010). Pinoresinol-lariciresinol reductases with opposite enantiospecificity determine the enantiomeric composition of lignans in the different organs of *Linum usitatissimum* L. *Planta Med.* 76 928–934. 10.1055/s-0030-1250036 20514607

[B23] Hölscher D. Schneider B. (2008). Application of laser-assisted microdissection for tissue and cell-specific analysis of RNA, proteins, and metabolites. *Prog. Bot.* 69 141–167. 10.1007/978-3-540-72954-9_6

[B24] Hölscher D. Shroff R. Knop K. Gottschaldt M. Crecelius A. Schneider B. (2009). Matrix-free UV-laser desorption/ionization (LDI) mass spectrometric imaging at the single-cell level: distribution of secondary metabolites of *Arabidopsis thaliana* and *Hypericum* species. *Plant J.* 60 907–918. 10.1111/j.1365-313X.2009.04012.x 19732382

[B25] Hu C. Y. Chee P. P. Chesney R. H. Zhou J. H. Miller P. D. O’brien W. T. (1990). Intrinsic GUS-like activities in seed plants. *Plant Cell Rep.* 9 1–5. 10.1007/BF00232123 24226366

[B26] Huis R. Morreel K. Fliniaux O. Lucau-Danila A. Fénart S. Grec S. (2012). Natural hypolignification is associated with extensive oligolignol accumulation in flax stems. *Plant Physiol.* 158 1893–1915. 10.1104/pp.111.192328 22331411 PMC3320194

[B27] Jefferson R. A. Kavanagh T. A. Bevan M. W. (1987). GUS fusions: β-glucuronidase as a sensitive and versatile gene fusion marker in higher plants. *EMBO J.* 6 3901–3907. 3327686 10.1002/j.1460-2075.1987.tb02730.x PMC553867

[B28] Kajiyama S. Harada K. Fukusaki E. Kobayashi A. (2006). Single cell-based analysis of *Torenia* petal pigments by a combination of ArF excimer laser micro sampling and nano-high performance liquid chromatography (HPLC)-mass spectrometry. *J. Biosci. Bioeng.* 102 575–578. 10.1263/jbb.102.575 17270726

[B29] Kamal-Eldin A. Peerlkamp N. Johnsson P. Andersson R. Andersson R. E. Lundgren L. N. (2001). An oligomer from flaxseed composed of secoisolariciresinoldiglucoside and 3-hydroxy-3-methyl glutaric acid residues. *Phytochemistry* 58 587–590. 10.1016/S0031-9422(01)00279-5 11576603

[B30] Kehr J. (2001). High resolution spatial analysis of plant systems. *Curr. Opin. Plant Biol.* 4 197–201. 10.1016/S1369-5266(00)00161-8 11312129

[B31] Kehr J. (2003). Single cell technology. *Curr. Opin. Plant Biol.* 6 617–621. 10.1016/j.pbi.2003.09.002 14611962

[B32] Kiyoto S. Yoshinaga A. Tanaka N. Wada M. Kamitakahara H. Takabe K. (2013). Immunolocalization of 8-5′ and 8-8′ linked structures of lignin in cell walls of *Chamaecyparis obtusa* using monoclonal antibodies. *Planta* 237 705–715. 10.1007/s00425-012-1784-x 23108661

[B33] Klosterman H. J. Smith F. (1954). The isolation of β-hydroxy-β-methylglutaric acid from the seed of flax (*Linum usitatissimum*). *J. Am. Chem. Soc.* 76 1229–1230. 10.1021/ja01634a007

[B34] Kosugi S. Ohashi Y. Nakajima K. Arai Y. (1990). An improved assay for β-glucuronidase in transformed cells: methanol almost completely suppresses a putative endogenous β-glucuronidase activity. *Plant Sci.* 70 133–140. 10.1016/0168-9452(90)90042-M

[B35] Kwon M. Davin L. B. Lewis N. G. (2001). In situ hybridization and immunolocalization of lignan reductases in woody tissues: implications for heartwood formation and other forms of vascular tissue preservation. *Phytochemistry* 57 899–914. 10.1016/S0031-9422(01)00108-X 11423140

[B36] Lapierre C. Monties B. Rolando C. (1986). Preparative thioacidolysis of spruce lignin – Isolation and identification of main monomeric products. *Holzforschung* 40 47–50. 10.1515/hfsg.1986.40.1.47

[B37] Li S. H. Schneider B. Gershenzon J. (2007). Microchemical analysis of laser-microdissected stone cells of Norway spruce by cryogenic nuclear magnetic resonance spectroscopy. *Planta* 225 771–779. 10.1007/s00425-006-0376-z 17039374

[B38] Madhusudhan B. Wiesenborn D. Schwarz J. Tostenson K. Gillespie J. (2000). A dry mechanical method for concentrating the lignan secoisolariciresinol diglucoside in flaxseed. *LWT Food Sci. Technol.* 33 268–275. 10.1006/fstl.2000.0652

[B39] Matas A. J. Yeats T. H. Buda G. J. Zheng Y. Chatterjee S. Tohge T. (2011). Tissue- and cell-type specific transcriptome profiling of expanding tomato fruit provides insights into metabolic and regulatory specialization and cuticle formation. *Plant Cell* 23 3893–3910. 10.1105/tpc.111.091173 22045915 PMC3246317

[B40] Moco S. Schneider B. Vervoort J. (2009). Plant micrometabolomics: the analysis of endogenous metabolites present in a plant cell or tissue. *J. Proteome Res.* 8 1694–1703. 10.1021/pr800973r 19714872

[B41] Morreel K. Ralph J. Kim H. Lu F. C. Goeminne G. Ralph S. (2004). Profiling of oligolignols reveals monolignol coupling conditions in lignifying poplar xylem. *Plant Physiol.* 136 3537–3549. 10.1104/pp.104.049304 15516504 PMC527153

[B42] Nakashima J. Chen F. Jackson L. Shadle G. Dixon R. A. (2008). Multi-site genetic modification of monolignol biosynthesis in alfalfa (*Medicago sativa*): effects on lignin composition in specific cell types. *New Phytol.* 179 738–750. 10.1111/j.1469-8137.2008.02502.x 18547377

[B43] Naran R. Chen G. B. Carpita N. C. (2008). Novel rhamnogalacturonan I and arabinoxylan polysaccharides of flax seed mucilage. *Plant Physiol.* 148 132–141. 10.1104/pp.108.123513 18667723 PMC2528086

[B44] Nelson T. Gandotra N. Tausta S. L. (2008). Plant cell types: reporting and sampling with new technologies. *Curr. Opin. Plant Biol.* 11 567–573. 10.1016/j.pbi.2008.06.006 18653377

[B45] Nelson T. Tausta S. L. Gandotra N. Liu T. (2006). Laser microdissection of plant tissue: what you see is what you get. *Annu. Rev. Plant Biol.* 57 181–201. 10.1146/annurev.arplant.56.032604.144138 16669760

[B46] Obel N. Erben V. Schwarz T. Kuhnel S. Fodor A. Pauly M. (2009). Microanalysis of plant cell wall polysaccharides. *Mol. Plant* 2 922–932. 10.1093/mp/ssp046 19825669

[B47] Olofsson L. Lundgren A. Brodelius P. E. (2012). Trichome isolation with and without fixation using laser microdissection and pressure catapulting followed by RNA amplification: expression of genes of terpene metabolism in apical and sub-apical trichome cells of *Artemisia annua* L. *Plant Sci.* 183 9–13. 10.1016/j.plantsci.2011.10.019 22195571

[B48] Oomah B. D. (2001). Flaxseed as a functional food source. *J. Sci. Food Agric.* 81 889–894. 10.1002/jsfa.898

[B49] Rajhi I. Yamauchi T. Takahashi H. Nishiuchi S. Shiono K. Watanabe R. (2011). Identification of genes expressed in maize root cortical cells during lysigenous aerenchyma formation using laser microdissection and microarray analyses. *New Phytol.* 190 351–368. 21091694 10.1111/j.1469-8137.2010.03535.x

[B50] Renouard S. Corbin C. Lopez T. Montguillon J. Gutierrez L. Lamblin F. (2012). Abscisic acid regulates pinoresinol–lariciresinol reductase gene expression and secoisolariciresinol accumulation in developing flax (*Linum usitatissimum* L.) *seeds*. *Planta* 235 85–98. 10.1007/s00425-011-1492-y 21837520

[B51] Renouard S. Tribalat M. A. Lamblin F. Mongelard G. Fliniaux O. Corbin C. (2014). RNAi-mediated pinoresinol lariciresinol reductase gene silencing in flax (*Linum usitatissimum*) seed coat : consequences on lignans and neolignans accumulation. *J. Plant Physiol.* 171 1372–1377. 10.1016/j.jplph.2014.06.005 25046758

[B52] Schiebold S. Tschiersch H. Borisjuk L. Heinzel N. Radchuk R. Rolletschek H. (2011). A novel procedure for the quantitative analysis of metabolites, storage products and transcripts of laser microdissected seed tissues of *Brassica napus*. *Plant Methods* 7:19 10.1186/1746-4811-7-19 PMC3141804 21718489

[B53] Schneider B. Hölscher D. (2007). Laser microdissection and cryogenic nuclear magnetic resonance spectroscopy: an alliance for cell type-specific metabolite profiling. *Planta* 225 763–770. 10.1007/s00425-006-0404-z 17006668

[B54] Simpson A. J. Brown S. A. (2005). Purge NMR: effective and easy solvent suppression. *J. Magn. Reson.* 175 340–346. 10.1016/j.jmr.2005.05.008 15964227

[B55] Singh K. K. Mridula D. Rehal J. Barnwal P. (2011). Flaxseed: a potential source of food, feed and fiber. *Crit. Rev. Food Sci. Nutr.* 51 210–222. 10.1080/10408390903537241 21390942

[B56] Struijs K. Vincken J. P. Doeswijk T. G. Voragen A. G. J. Gruppen H. (2009). The chain length of lignan macromolecule from flaxseed hulls is determined by the incorporation of coumaric acid glucosides and ferulic acid glucosides. *Phytochemistry* 70 262–269. 10.1016/j.phytochem.2008.12.015 19155025

[B57] Svatoš A. (2011). Single-cell metabolomics comes of age: new developments in mass spectrometry profiling and imaging. *Anal. Chem.* 83 5037–5044. 10.1021/ac2003592 21630635

[B58] Thiel J. Mueller M. Weschke W. Weber H. (2009). Amino acid metabolism at the maternal-filial boundary of young barley seeds: a microdissection-based study. *Planta* 230 205–213. 10.1007/s00425-009-0935-1 19415324

[B59] Thompson L. U. Robb P. Serraino M. Cheung F. (1991). Mammalian lignan production from various foods. *Nutr. Cancer* 16 43–52. 10.1080/01635589109514139 1656395

[B60] Tokunaga N. Sakakibara N. Umezawa T. Ito Y. Fukuda H. Sato Y. (2005). Involvement of extracellular dilignols in lignification during tracheary element differentiation of isolated *Zinnia* mesophyll cells. *Plant Cell Physiol.* 46 224–232. 10.1093/pcp/pci017 15659440

[B61] Umezawa T. Davin L. B. Lewis N. G. (1991). Formation of lignans (-)-secoisolariciresinol and (-)-matairesinol with *Forsythia intermedia* cell-free extracts. *J. Biol. Chem.* 266 10210–10217. 2037574

[B62] Wiesenborn D. Tostenson K. Kangas N. (2003). Continuous abrasive method for mechanically fractionating flaxseed. *J. Am. Oil Chem. Soc.* 80 295–300. 10.1007/s11746-003-0692-2

[B63] Willför S. M. Ahotupa M. O. Hemming J. E. Reunanen M. H. T. Eklund P. C. Sjöholm R. E. (2003). Antioxidant activity of knotwood extractives and phenolic compounds of selected tree species. *J. Agric. Food Chem.* 51 7600–7606. 10.1021/jf030445h 14664514

[B64] Zhang W. B. Xu S. Y. Wang Z. Yang R. J. Lu R. R. (2009). Demucilaging and dehulling flaxseed with a wet process. *LWT Food Sci. Technol.* 42 1193–1198. 10.1016/j.lwt.2009.01.001

[B65] Zhao Q. Zeng Y. Yin Y. Pu Y. Jackson L. A. Engle N. L. (2015). Pinoresinol reductase 1 impacts lignin distribution during secondary cell wall biosynthesis in *Arabidopsis*. *Phytochemistry* 112 170–178. 10.1016/j.phytochem.2014.07.008 25107662

