# Supplementary material for: Laser Microdissection and Spatiotemporal Pinoresinol-Lariciresinol Reductase Gene Expression Assign the Cell Layer-Specific Accumulation of Secoisolariciresinol Diglucoside in Flaxseed Coats
Source: Front Plant Sci. 2016 Nov 21;7:1743. doi: 10.3389/fpls.2016.01743 (PMC5116464; doi:10.3389/fpls.2016.01743)
Supplement: Supplementary file 1 [file Image_1.PDF]

Supplementary material

Fig. S1. Mechanical separation of the flaxseed coats. (A) Picture of the flaxseed coats; (B) flaxseed coats separated into two parts **A** and **B**; (C) some mucilageneous cell (MC) material was isolated from seed coats as part **C**. Bars in A and B represent 1.0 mm, and the bar in C represents 0.5 mm.

Fig. S2. HPLC chromatograms of alkaline hydrolysis of separated samples. Chromatograms **A**, **B**, **C**, **B1**, and **B2** were obtained from mature seed samples. **SDG**: Chromatogram of secoisolariciresinol diglucoside (SDG). Chromatograms **I**, **II**, and **III** were obtained from 25 DAF seed samples. For details of sample preparation, see Figures 1 and 2. Framed peaks in different chromatograms represent SDG. Some peaks in chromatogram **B** were identified by comparing with standards as 4-(β-D-glucopyranosyl) coumarate (1), 4-(β-D-glucopyranosyl) ferulate (2), methyl 4-(β-D-glucopyranosyl) caffeate (3), methyl 4-(β-D-glucopyranosyl) coumarate (4), and methyl 4-(β-D-glucopyranosyl) ferulate (5).

Fig. S1.

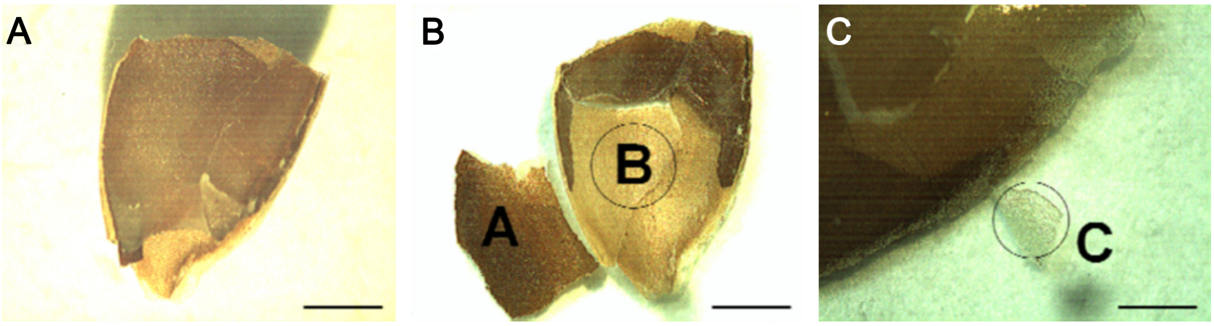

Fig. S2.

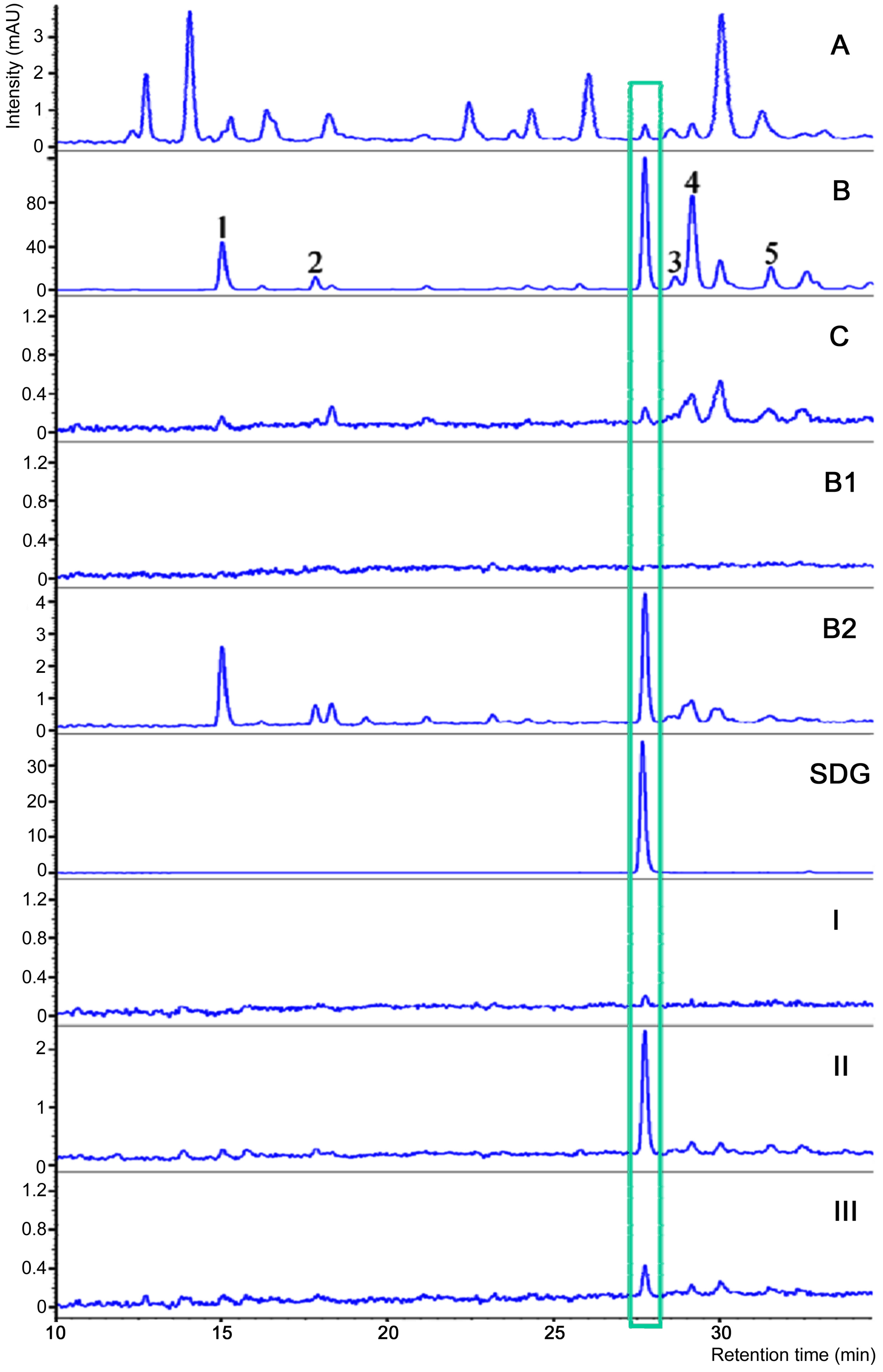

Intensity (mAU)
A
B
C
B1
B2
SDG
I
II
III
1
2
3
4
5
Retention time (min)
10
15
20
25
30
